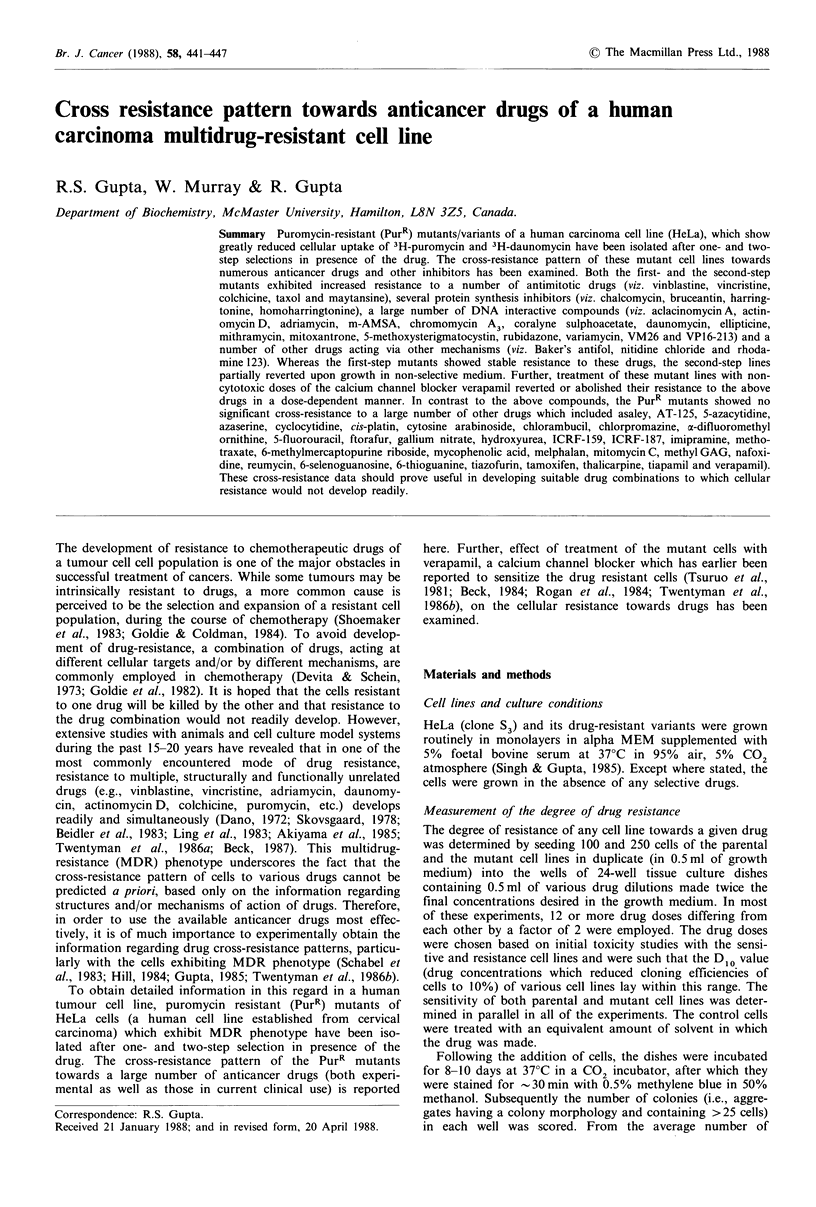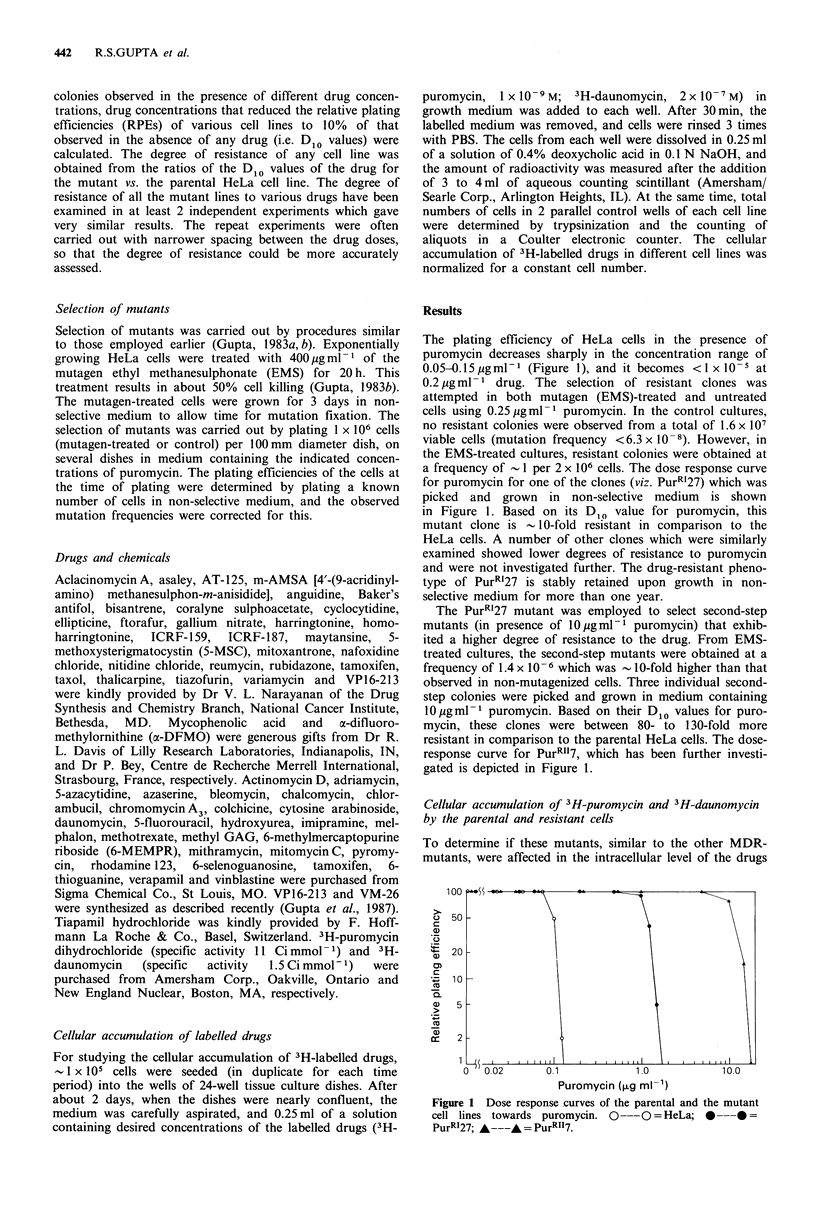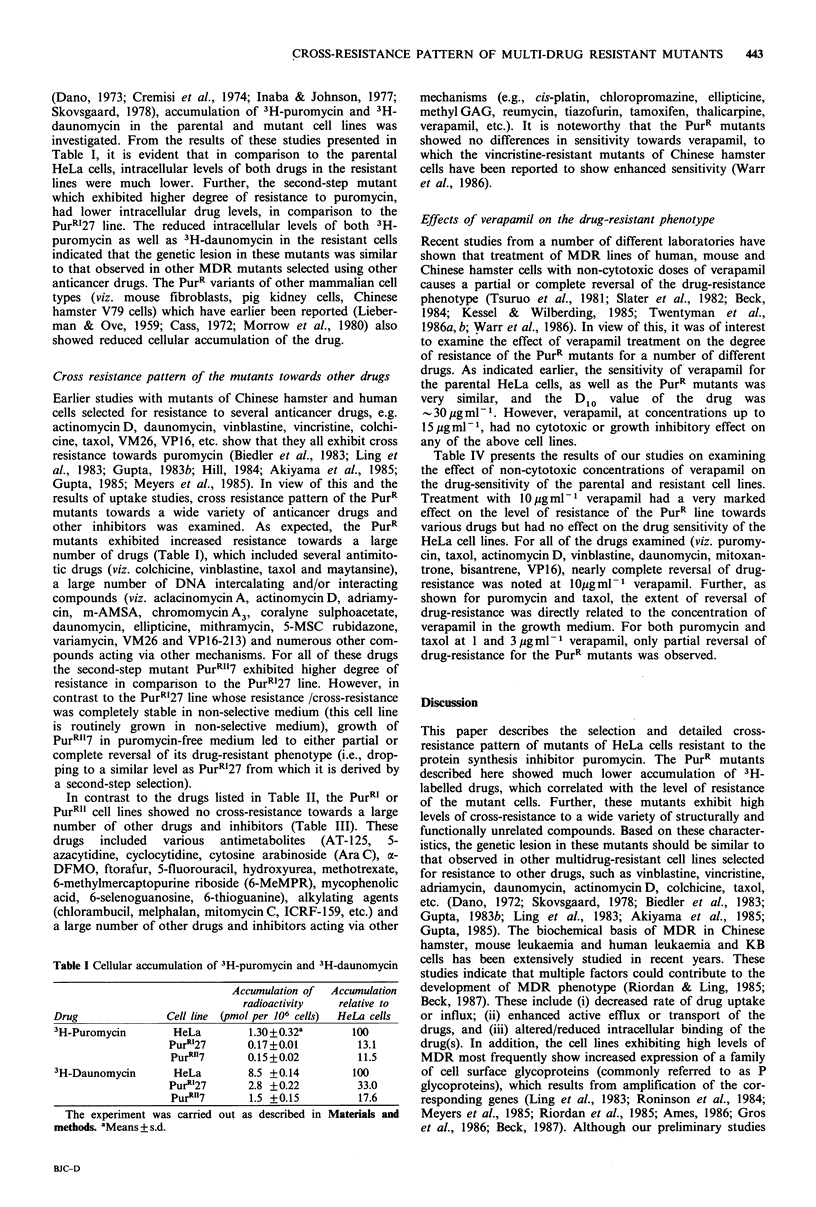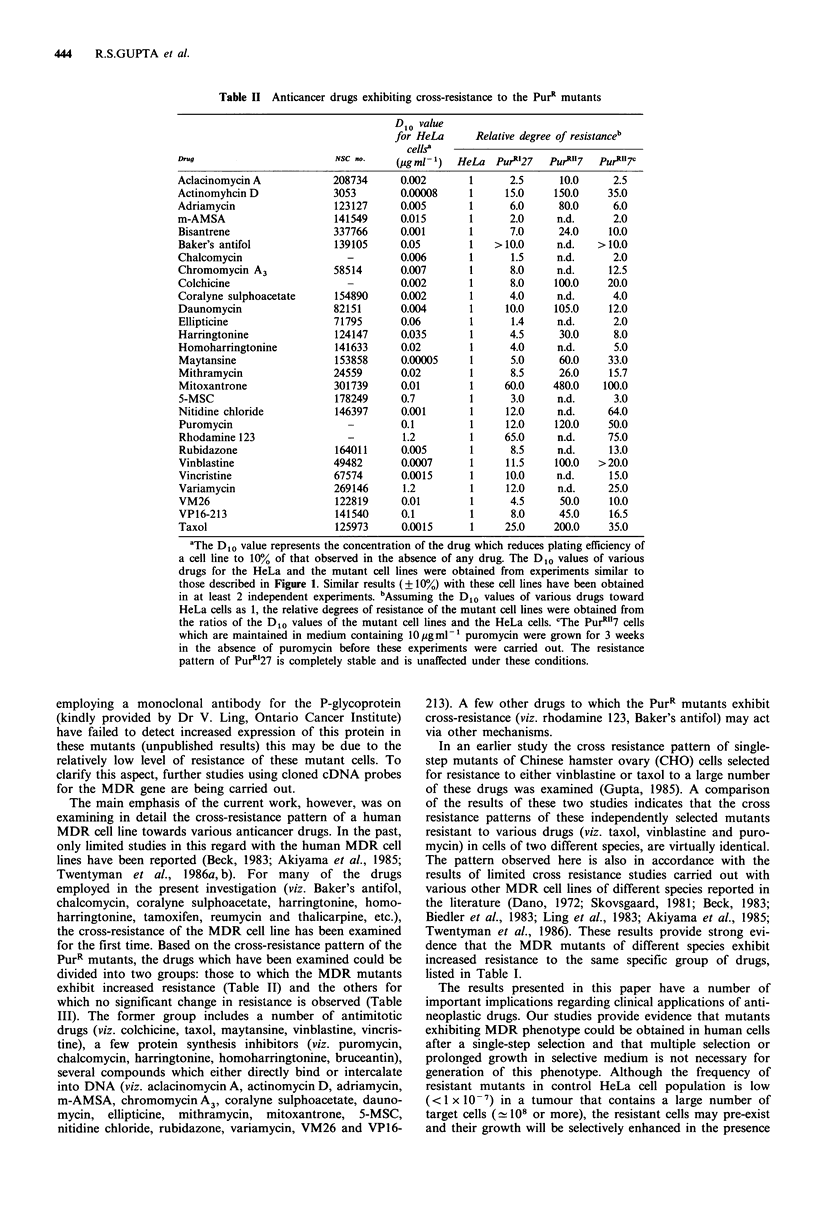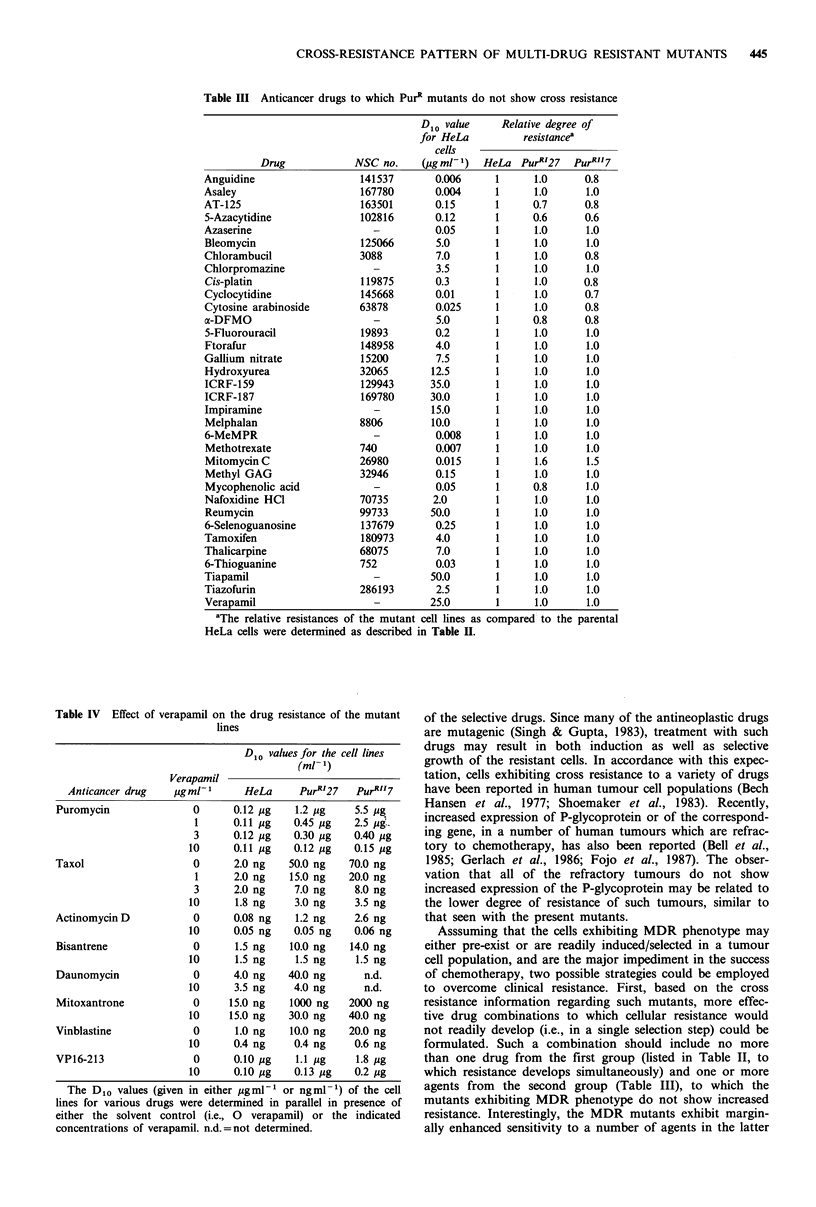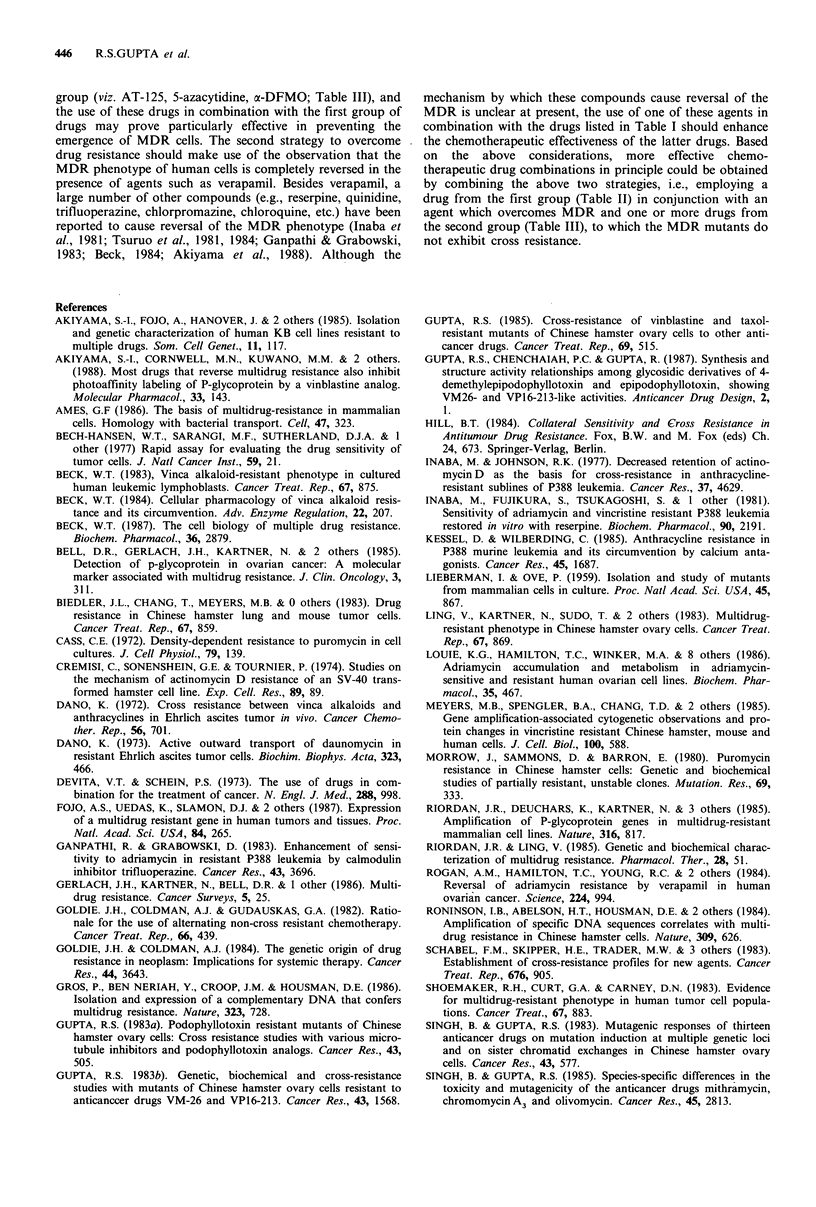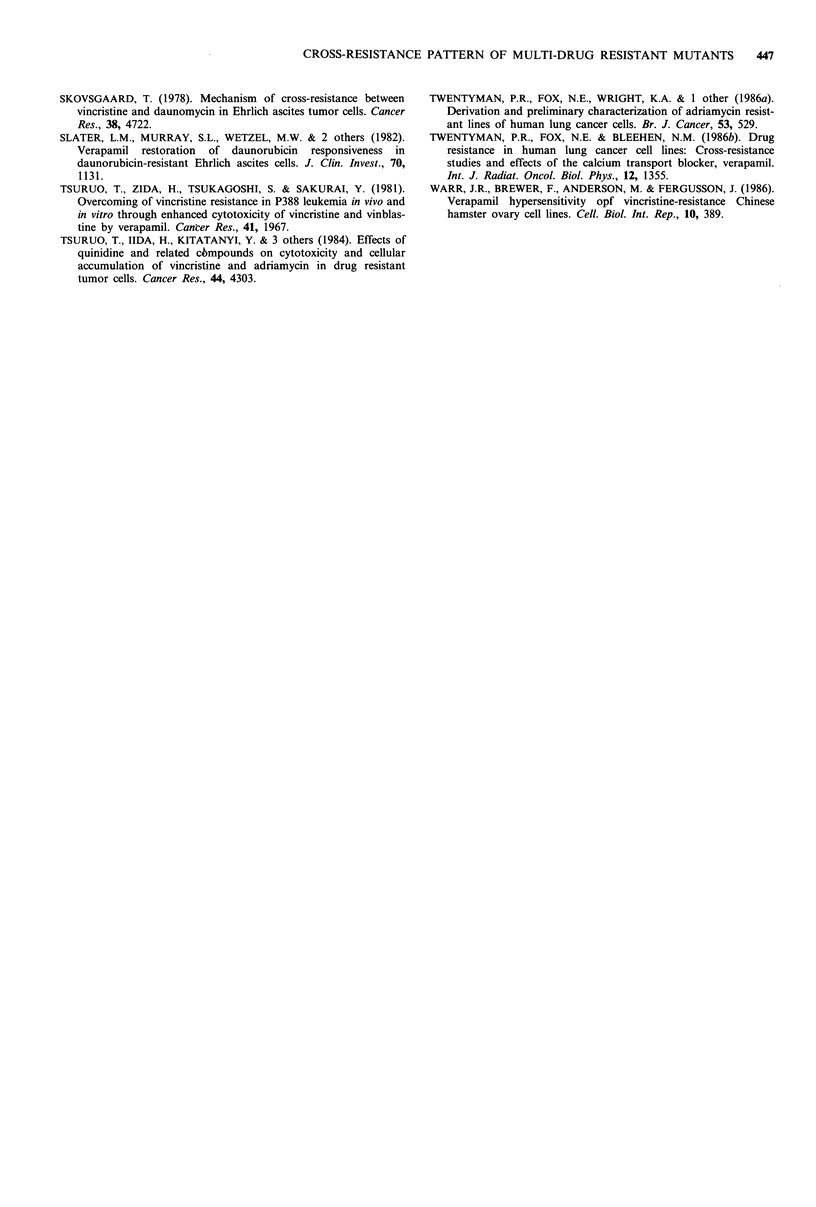# Cross resistance pattern towards anticancer drugs of a human carcinoma multidrug-resistant cell line.

**DOI:** 10.1038/bjc.1988.237

**Published:** 1988-10

**Authors:** R. S. Gupta, W. Murray, R. Gupta

**Affiliations:** Department of Biochemistry, McMaster University, Hamilton, Canada.

## Abstract

Puromycin-resistant (PurR) mutants/variants of a human carcinoma cell line (HeLa), which show greatly reduced cellular uptake of 3H-puromycin and 3H-daunomycin have been isolated after one- and two-step selections in presence of the drug. The cross-resistance pattern of these mutant cell lines towards numerous anticancer drugs and other inhibitors has been examined. Both the first- and the second-step mutants exhibited increased resistance to a number of antimitotic drugs (viz. vinblastine, vincristine, colchicine, taxol and maytansine), several protein synthesis inhibitors (viz. chalcomycin, bruceantin, harringtonine, homoharringtonine), a large number of DNA interactive compounds (viz. aclacinomycin A, actinomycin D, adriamycin, m-AMSA, chromomycin A3, coralyne sulphoacetate, daunomycin, ellipticine, mithramycin, mitoxantrone, 5-methoxysterigmatocystin, rubidazone, variamycin, VM26 and VP16-213) and a number of other drugs acting via other mechanisms (viz. Baker's antifol, nitidine chloride and rhodamine 123). Whereas the first-step mutants showed stable resistance to these drugs, the second-step lines partially reverted upon growth in non-selective medium. Further, treatment of these mutant lines with non-cytotoxic doses of the calcium channel blocker verapamil reverted or abolished their resistance to the above drugs in a dose-dependent manner. In contrast to the above compounds, the PurR mutants showed no significant cross-resistance to a large number of other drugs which included asaley, AT-125, 5-azacytidine, azaserine, cyclocytidine, cis-platin, cytosine arabinoside, chlorambucil, chlorpromazine, alpha-difluoromethyl ornithine, 5-fluorouracil, ftorafur, gallium nitrate, hydroxyurea, ICRF-159, ICRF-187, imipramine, methotraxate, 6-methylmercaptopurine riboside, mycophenolic acid, melphalan, mitomycin C, methyl GAG, nafoxidine, reumycin, 6-selenoguanosine, 6-thioguanine, tiazofurin, tamoxifen, thalicarpine, tiapamil and verapamil). These cross-resistance data should prove useful in developing suitable drug combinations to which cellular resistance would not develop readily.


*Br. J. Cancer* (1988), **58**, 441–447 © The Macmillan Press Ltd., 1988

### Cross resistance pattern towards anticancer drugs of a human carcinoma multidrug-resistant cell line

R.S. Gupta, W. Murray & R. Gupta

*Department of Biochemistry, McMaster University, Hamilton, L8N 3Z5, Canada.*

**Summary** Puromycin-resistant ($Pur^R$) mutants/variants of a human carcinoma cell line (HeLa), which show greatly reduced cellular uptake of $^3$H-puromycin and $^3$H-daunomycin have been isolated after one- and two-step selections in presence of the drug. The cross-resistance pattern of these mutant cell lines towards numerous anticancer drugs and other inhibitors has been examined. Both the first- and the second-step mutants exhibited increased resistance to a number of antimitotic drugs (*viz.* vinblastine, vincristine, colchicine, taxol and maytansine), several protein synthesis inhibitors (*viz.* chalcomycin, bruceantin, harringtonine, homoharringtonine), a large number of DNA interactive compounds (*viz.* aclacinomycin A, actinomycin D, adriamycin, m-AMSA, chromomycin $A_3$, coralyne sulphoacetate, daunomycin, ellipticine, mithramycin, mitoxantrone, 5-methoxysterigmatocystin, rubidazone, variamycin, VM26 and VP16-213) and a number of other drugs acting via other mechanisms (*viz.* Baker's antifol, nitidine chloride and rhodamine 123). Whereas the first-step mutants showed stable resistance to these drugs, the second-step lines partially reverted upon growth in non-selective medium. Further, treatment of these mutant lines with non-cytotoxic doses of the calcium channel blocker verapamil reverted or abolished their resistance to the above drugs in a dose-dependent manner. In contrast to the above compounds, the $Pur^R$ mutants showed no significant cross-resistance to a large number of other drugs which included asaley, AT-125, 5-azacytidine, azaserine, cyclocytidine, *cis*-platin, cytosine arabinoside, chlorambucil, chlorpromazine, α-difluoromethyl ornithine, 5-fluorouracil, ftorafur, gallium nitrate, hydroxyurea, ICRF-159, ICRF-187, imipramine, methotraxate, 6-methylmercaptopurine riboside, mycophenolic acid, melphalan, mitomycin C, methyl GAG, nafoxidine, reumycin, 6-selenoguanosine, 6-thioguanine, tiazofurin, tamoxifen, thalicarpine, tiapamil and verapamil). These cross-resistance data should prove useful in developing suitable drug combinations to which cellular resistance would not develop readily.

The development of resistance to chemotherapeutic drugs of a tumour cell cell population is one of the major obstacles in successful treatment of cancers. While some tumours may be intrinsically resistant to drugs, a more common cause is perceived to be the selection and expansion of a resistant cell population, during the course of chemotherapy (Shoemaker *et al.*, 1983; Goldie & Coldman, 1984). To avoid development of drug-resistance, a combination of drugs, acting at different cellular targets and/or by different mechanisms, are commonly employed in chemotherapy (Devita & Schein, 1973; Goldie *et al.*, 1982). It is hoped that the cells resistant to one drug will be killed by the other and that resistance to the drug combination would not readily develop. However, extensive studies with animals and cell culture model systems during the past 15–20 years have revealed that in one of the most commonly encountered mode of drug resistance, resistance to multiple, structurally and functionally unrelated drugs (e.g., vinblastine, vincristine, adriamycin, daunomycin, actinomycin D, colchicine, puromycin, etc.) develops readily and simultaneously (Dano, 1972; Skovsgaard, 1978; Beidler *et al.*, 1983; Ling *et al.*, 1983; Akiyama *et al.*, 1985; Twentyman *et al.*, 1986*a*; Beck, 1987). This multidrug-resistance (MDR) phenotype underscores the fact that the cross-resistance pattern of cells to various drugs cannot be predicted *a priori*, based only on the information regarding structures and/or mechanisms of action of drugs. Therefore, in order to use the available anticancer drugs most effectively, it is of much importance to experimentally obtain the information regarding drug cross-resistance patterns, particularly with the cells exhibiting MDR phenotype (Schabel *et al.*, 1983; Hill, 1984; Gupta, 1985; Twentyman *et al.*, 1986*b*).

To obtain detailed information in this regard in a human tumour cell line, puromycin resistant ($Pur^R$) mutants of HeLa cells (a human cell line established from cervical carcinoma) which exhibit MDR phenotype have been isolated after one- and two-step selection in presence of the drug. The cross-resistance pattern of the $Pur^R$ mutants towards a large number of anticancer drugs (both experimental as well as those in current clinical use) is reported here. Further, effect of treatment of the mutant cells with verapamil, a calcium channel blocker which has earlier been reported to sensitize the drug resistant cells (Tsuruo *et al.*, 1981; Beck, 1984; Rogan *et al.*, 1984; Twentyman *et al.*, 1986*b*), on the cellular resistance towards drugs has been examined.

Correspondence: R.S. Gupta.
Received 21 January 1988; and in revised form, 20 April 1988.

### Materials and methods

### *Cell lines and culture conditions*

HeLa (clone $S_3$) and its drug-resistant variants were grown routinely in monolayers in alpha MEM supplemented with 5% foetal bovine serum at 37°C in 95% air, 5% $CO_2$ atmosphere (Singh & Gupta, 1985). Except where stated, the cells were grown in the absence of any selective drugs.

### *Measurement of the degree of drug resistance*

The degree of resistance of any cell line towards a given drug was determined by seeding 100 and 250 cells of the parental and the mutant cell lines in duplicate (in 0.5 ml of growth medium) into the wells of 24-well tissue culture dishes containing 0.5 ml of various drug dilutions made twice the final concentrations desired in the growth medium. In most of these experiments, 12 or more drug doses differing from each other by a factor of 2 were employed. The drug doses were chosen based on initial toxicity studies with the sensitive and resistance cell lines and were such that the $D_{10}$ value (drug concentrations which reduced cloning efficiencies of cells to 10%) of various cell lines lay within this range. The sensitivity of both parental and mutant cell lines was determined in parallel in all of the experiments. The control cells were treated with an equivalent amount of solvent in which the drug was made.

Following the addition of cells, the dishes were incubated for 8–10 days at 37°C in a $CO_2$ incubator, after which they were stained for ~30 min with 0.5% methylene blue in 50% methanol. Subsequently the number of colonies (i.e., aggregates having a colony morphology and containing >25 cells) in each well was scored. From the average number of colonies observed in the presence of different drug concentrations, drug concentrations that reduced the relative plating efficiencies (RPEs) of various cell lines to 10% of that observed in the absence of any drug (i.e. $D_{10}$ values) were calculated. The degree of resistance of any cell line was obtained from the ratios of the $D_{10}$ values of the drug for the mutant *vs.* the parental HeLa cell line. The degree of resistance of all the mutant lines to various drugs have been examined in at least 2 independent experiments which gave very similar results. The repeat experiments were often carried out with narrower spacing between the drug doses, so that the degree of resistance could be more accurately assessed.

*Selection of mutants*

Selection of mutants was carried out by procedures similar to those employed earlier (Gupta, 1983*a*, *b*). Exponentially growing HeLa cells were treated with $400\,\mu g\,ml^{-1}$ of the mutagen ethyl methanesulphonate (EMS) for 20 h. This treatment results in about 50% cell killing (Gupta, 1983*b*). The mutagen-treated cells were grown for 3 days in non-selective medium to allow time for mutation fixation. The selection of mutants was carried out by plating $1 \times 10^6$ cells (mutagen-treated or control) per 100 mm diameter dish, on several dishes in medium containing the indicated concentrations of puromycin. The plating efficiencies of the cells at the time of plating were determined by plating a known number of cells in non-selective medium, and the observed mutation frequencies were corrected for this.

*Drugs and chemicals*

Aclacinomycin A, asaley, AT-125, m-AMSA [4′-(9-acridinylamino) methanesulphon-*m*-anisidide], anguidine, Baker's antifol, bisantrene, coralyne sulphoacetate, cyclocytidine, ellipticine, ftorafur, gallium nitrate, harringtonine, homoharringtonine, ICRF-159, ICRF-187, maytansine, 5-methoxysterigmatocystin (5-MSC), mitoxantrone, nafoxidine chloride, nitidine chloride, reumycin, rubidazone, tamoxifen, taxol, thalicarpine, tiazofurin, variamycin and VP16-213 were kindly provided by Dr V. L. Narayanan of the Drug Synthesis and Chemistry Branch, National Cancer Institute, Bethesda, MD. Mycophenolic acid and α-difluoromethylornithine (α-DFMO) were generous gifts from Dr R. L. Davis of Lilly Research Laboratories, Indianapolis, IN, and Dr P. Bey, Centre de Recherche Merrell International, Strasbourg, France, respectively. Actinomycin D, adriamycin, 5-azacytidine, azaserine, bleomycin, chalcomycin, chlorambucil, chromomycin $A_3$, colchicine, cytosine arabinoside, daunomycin, 5-fluorouracil, hydroxyurea, imipramine, melphalon, methotrexate, methyl GAG, 6-methylmercaptopurine riboside (6-MEMPR), mithramycin, mitomycin C, pyromycin, rhodamine 123, 6-selenoguanosine, tamoxifen, 6-thioguanine, verapamil and vinblastine were purchased from Sigma Chemical Co., St Louis, MO. VP16-213 and VM-26 were synthesized as described recently (Gupta *et al.*, 1987). Tiapamil hydrochloride was kindly provided by F. Hoffmann La Roche & Co., Basel, Switzerland. $^3$H-puromycin dihydrochloride (specific activity 11 Ci mmol$^{-1}$) and $^3$H-daunomycin (specific activity 1.5 Ci mmol$^{-1}$) were purchased from Amersham Corp., Oakville, Ontario and New England Nuclear, Boston, MA, respectively.

*Cellular accumulation of labelled drugs*

For studying the cellular accumulation of $^3$H-labelled drugs, $\sim 1 \times 10^5$ cells were seeded (in duplicate for each time period) into the wells of 24-well tissue culture dishes. After about 2 days, when the dishes were nearly confluent, the medium was carefully aspirated, and 0.25 ml of a solution containing desired concentrations of the labelled drugs ($^3$H-puromycin, $1 \times 10^{-9}$ M; $^3$H-daunomycin, $2 \times 10^{-7}$ M) in growth medium was added to each well. After 30 min, the labelled medium was removed, and cells were rinsed 3 times with PBS. The cells from each well were dissolved in 0.25 ml of a solution of 0.4% deoxycholic acid in 0.1 N NaOH, and the amount of radioactivity was measured after the addition of 3 to 4 ml of aqueous counting scintillant (Amersham/Searle Corp., Arlington Heights, IL). At the same time, total numbers of cells in 2 parallel control wells of each cell line were determined by trypsinization and the counting of aliquots in a Coulter electronic counter. The cellular accumulation of $^3$H-labelled drugs in different cell lines was normalized for a constant cell number.

### Results

The plating efficiency of HeLa cells in the presence of puromycin decreases sharply in the concentration range of $0.05–0.15\,\mu g\,ml^{-1}$ (Figure 1), and it becomes $<1 \times 10^{-5}$ at $0.2\,\mu g\,ml^{-1}$ drug. The selection of resistant clones was attempted in both mutagen (EMS)-treated and untreated cells using $0.25\,\mu g\,ml^{-1}$ puromycin. In the control cultures, no resistant colonies were observed from a total of $1.6 \times 10^7$ viable cells (mutation frequency $<6.3 \times 10^{-8}$). However, in the EMS-treated cultures, resistant colonies were obtained at a frequency of $\sim 1$ per $2 \times 10^6$ cells. The dose response curve for puromycin for one of the clones (*viz.* Pur$^{RI}$27) which was picked and grown in non-selective medium is shown in Figure 1. Based on its $D_{10}$ value for puromycin, this mutant clone is $\sim 10$-fold resistant in comparison to the HeLa cells. A number of other clones which were similarly examined showed lower degrees of resistance to puromycin and were not investigated further. The drug-resistant phenotype of Pur$^{RI}$27 is stably retained upon growth in non-selective medium for more than one year.

The Pur$^{RI}$27 mutant was employed to select second-step mutants (in presence of $10\,\mu g\,ml^{-1}$ puromycin) that exhibited a higher degree of resistance to the drug. From EMS-treated cultures, the second-step mutants were obtained at a frequency of $1.4 \times 10^{-6}$ which was $\sim 10$-fold higher than that observed in non-mutagenized cells. Three individual second-step colonies were picked and grown in medium containing $10\,\mu g\,ml^{-1}$ puromycin. Based on their $D_{10}$ values for puromycin, these clones were between 80- to 130-fold more resistant in comparison to the parental HeLa cells. The dose-response curve for Pur$^{RII}$7, which has been further investigated is depicted in Figure 1.

*Cellular accumulation of $^3$H-puromycin and $^3$H-daunomycin by the parental and resistant cells*

To determine if these mutants, similar to the other MDR-mutants, were affected in the intracellular level of the drugs

**Figure 1** Dose response curves of the parental and the mutant cell lines towards puromycin. ○---○ = HeLa; ●---● = Pur$^{RI}$27; ▲---▲ = Pur$^{RII}$7.

(Dano, 1973; Cremisi *et al.*, 1974; Inaba & Johnson, 1977; Skovsgaard, 1978), accumulation of $^3$H-puromycin and $^3$H-daunomycin in the parental and mutant cell lines was investigated. From the results of these studies presented in Table I, it is evident that in comparison to the parental HeLa cells, intracellular levels of both drugs in the resistant lines were much lower. Further, the second-step mutant which exhibited higher degree of resistance to puromycin, had lower intracellular drug levels, in comparison to the Pur$^{RI}$27 line. The reduced intracellular levels of both $^3$H-puromycin as well as $^3$H-daunomycin in the resistant cells indicated that the genetic lesion in these mutants was similar to that observed in other MDR mutants selected using other anticancer drugs. The Pur$^R$ variants of other mammalian cell types (*viz.* mouse fibroblasts, pig kidney cells, Chinese hamster V79 cells) which have earlier been reported (Lieberman & Ove, 1959; Cass, 1972; Morrow *et al.*, 1980) also showed reduced cellular accumulation of the drug.

*Cross resistance pattern of the mutants towards other drugs*

Earlier studies with mutants of Chinese hamster and human cells selected for resistance to several anticancer drugs, e.g. actinomycin D, daunomycin, vinblastine, vincristine, colchicine, taxol, VM26, VP16, etc. show that they all exhibit cross resistance towards puromycin (Biedler *et al.*, 1983; Ling *et al.*, 1983; Gupta, 1983*b*; Hill, 1984; Akiyama *et al.*, 1985; Gupta, 1985; Meyers *et al.*, 1985). In view of this and the results of uptake studies, cross resistance pattern of the Pur$^R$ mutants towards a wide variety of anticancer drugs and other inhibitors was examined. As expected, the Pur$^R$ mutants exhibited increased resistance towards a large number of drugs (Table I), which included several antimitotic drugs (*viz.* colchicine, vinblastine, taxol and maytansine), a large number of DNA intercalating and/or interacting compounds (*viz.* aclacinomycin A, actinomycin D, adriamycin, m-AMSA, chromomycin $A_3$, coralyne sulphoacetate, daunomycin, ellipticine, mithramycin, 5-MSC rubidazone, variamycin, VM26 and VP16-213) and numerous other compounds acting via other mechanisms. For all of these drugs the second-step mutant Pur$^{RII}$7 exhibited higher degree of resistance in comparison to the Pur$^{RI}$27 line. However, in contrast to the Pur$^{RI}$27 line whose resistance /cross-resistance was completely stable in non-selective medium (this cell line is routinely grown in non-selective medium), growth of Pur$^{RII}$7 in puromycin-free medium led to either partial or complete reversal of its drug-resistant phenotype (i.e., dropping to a similar level as Pur$^{RI}$27 from which it is derived by a second-step selection).

In contrast to the drugs listed in Table II, the Pur$^{RI}$ or Pur$^{RII}$ cell lines showed no cross-resistance towards a large number of other drugs and inhibitors (Table III). These drugs included various antimetabolites (AT-125, 5-azacytidine, cyclocytidine, cytosine arabinoside (Ara C), α-DFMO, ftorafur, 5-fluorouracil, hydroxyurea, methotrexate, 6-methylmercaptopurine riboside (6-MeMPR), mycophenolic acid, 6-selenoguanosine, 6-thioguanine), alkylating agents (chlorambucil, melphalan, mitomycin C, ICRF-159, etc.) and a large number of other drugs and inhibitors acting via other mechanisms (e.g., *cis*-platin, chloropromazine, ellipticine, methyl GAG, reumycin, tiazofurin, tamoxifen, thalicarpine, verapamil, etc.). It is noteworthy that the Pur$^R$ mutants showed no differences in sensitivity towards verapamil, to which the vincristine-resistant mutants of Chinese hamster cells have been reported to show enhanced sensitivity (Warr *et al.*, 1986).

**Table I** Cellular accumulation of $^3$H-puromycin and $^3$H-daunomycin

| *Drug* | *Cell line* | *Accumulation of radioactivity (pmol per 10$^6$ cells)* | *Accumulation relative to HeLa cells* |
|---|---|---|---|
| $^3$H-Puromycin | HeLa | 1.30±0.32$^a$ | 100 |
| | Pur$^{RI}$27 | 0.17±0.01 | 13.1 |
| | Pur$^{RII}$7 | 0.15±0.02 | 11.5 |
| $^3$H-Daunomycin | HeLa | 8.5 ±0.14 | 100 |
| | Pur$^{RI}$27 | 2.8 ±0.22 | 33.0 |
| | Pur$^{RII}$7 | 1.5 ±0.15 | 17.6 |

The experiment was carried out as described in **Materials and methods.** $^a$Means±s.d.

*Effects of verapamil on the drug-resistant phenotype*

Recent studies from a number of different laboratories have shown that treatment of MDR lines of human, mouse and Chinese hamster cells with non-cytotoxic doses of verapamil causes a partial or complete reversal of the drug-resistance phenotype (Tsuruo *et al.*, 1981; Slater *et al.*, 1982; Beck, 1984; Kessel & Wilberding, 1985; Twentyman *et al.*, 1986*a*, *b*; Warr *et al.*, 1986). In view of this, it was of interest to examine the effect of verapamil treatment on the degree of resistance of the Pur$^R$ mutants for a number of different drugs. As indicated earlier, the sensitivity of verapamil for the parental HeLa cells, as well as the Pur$^R$ mutants was very similar, and the $D_{10}$ value of the drug was $\sim 30\,\mu g\,ml^{-1}$. However, verapamil, at concentrations up to $15\,\mu g\,ml^{-1}$, had no cytotoxic or growth inhibitory effect on any of the above cell lines.

Table IV presents the results of our studies on examining the effect of non-cytotoxic concentrations of verapamil on the drug-sensitivity of the parental and resistant cell lines. Treatment with $10\,\mu g\,ml^{-1}$ verapamil had a very marked effect on the level of resistance of the Pur$^R$ line towards various drugs but had no effect on the drug sensitivity of the HeLa cell lines. For all of the drugs examined (*viz.* puromycin, taxol, actinomycin D, vinblastine, daunomycin, mitoxantrone, bisantrene, VP16), nearly complete reversal of drug-resistance was noted at $10\mu g\,ml^{-1}$ verapamil. Further, as shown for puromycin and taxol, the extent of reversal of drug-resistance was directly related to the concentration of verapamil in the growth medium. For both puromycin and taxol at 1 and $3\,\mu g\,ml^{-1}$ verapamil, only partial reversal of drug-resistance for the Pur$^R$ mutants was observed.

### Discussion

This paper describes the selection and detailed cross-resistance pattern of mutants of HeLa cells resistant to the protein synthesis inhibitor puromycin. The Pur$^R$ mutants described here showed much lower accumulation of $^3$H-labelled drugs, which correlated with the level of resistance of the mutant cells. Further, these mutants exhibit high levels of cross-resistance to a wide variety of structurally and functionally unrelated compounds. Based on these characteristics, the genetic lesion in these mutants should be similar to that observed in other multidrug-resistant cell lines selected for resistance to other drugs, such as vinblastine, vincristine, adriamycin, daunomycin, actinomycin D, colchicine, taxol, etc. (Dano, 1972; Skovsgaard, 1978; Biedler *et al.*, 1983; Gupta, 1983*b*; Ling *et al.*, 1983; Akiyama *et al.*, 1985; Gupta, 1985). The biochemical basis of MDR in Chinese hamster, mouse leukaemia and human leukaemia and KB cells has been extensively studied in recent years. These studies indicate that multiple factors could contribute to the development of MDR phenotype (Riordan & Ling, 1985; Beck, 1987). These include (i) decreased rate of drug uptake or influx; (ii) enhanced active efflux or transport of the drugs, and (iii) altered/reduced intracellular binding of the drug(s). In addition, the cell lines exhibiting high levels of MDR most frequently show increased expression of a family of cell surface glycoproteins (commonly referred to as P glycoproteins), which results from amplification of the corresponding genes (Ling *et al.*, 1983; Roninson *et al.*, 1984; Meyers *et al.*, 1985; Riordan *et al.*, 1985; Ames, 1986; Gros *et al.*, 1986; Beck, 1987). Although our preliminary studies

**Table II** Anticancer drugs exhibiting cross-resistance to the $Pur^R$ mutants

| *Drug* | *NSC no.* | $D_{10}$ *value for HeLa cells*[a] ($\mu g\,ml^{-1}$) | *Relative degree of resistance*[b] | | | |
|---|---|---|---|---|---|---|
| | | | *HeLa* | $Pur^{RI}27$ | $Pur^{RII}7$ | $Pur^{RII}7$[c] |
| Aclacinomycin A | 208734 | 0.002 | 1 | 2.5 | 10.0 | 2.5 |
| Actinomyhcin D | 3053 | 0.00008 | 1 | 15.0 | 150.0 | 35.0 |
| Adriamycin | 123127 | 0.005 | 1 | 6.0 | 80.0 | 6.0 |
| m-AMSA | 141549 | 0.015 | 1 | 2.0 | n.d. | 2.0 |
| Bisantrene | 337766 | 0.001 | 1 | 7.0 | 24.0 | 10.0 |
| Baker's antifol | 139105 | 0.05 | 1 | >10.0 | n.d. | >10.0 |
| Chalcomycin | – | 0.006 | 1 | 1.5 | n.d. | 2.0 |
| Chromomycin $A_3$ | 58514 | 0.007 | 1 | 8.0 | n.d. | 12.5 |
| Colchicine | – | 0.002 | 1 | 8.0 | 100.0 | 20.0 |
| Coralyne sulphoacetate | 154890 | 0.002 | 1 | 4.0 | n.d. | 4.0 |
| Daunomycin | 82151 | 0.004 | 1 | 10.0 | 105.0 | 12.0 |
| Ellipticine | 71795 | 0.06 | 1 | 1.4 | n.d. | 2.0 |
| Harringtonine | 124147 | 0.035 | 1 | 4.5 | 30.0 | 8.0 |
| Homoharringtonine | 141633 | 0.02 | 1 | 4.0 | n.d. | 5.0 |
| Maytansine | 153858 | 0.00005 | 1 | 5.0 | 60.0 | 33.0 |
| Mithramycin | 24559 | 0.02 | 1 | 8.5 | 26.0 | 15.7 |
| Mitoxantrone | 301739 | 0.01 | 1 | 60.0 | 480.0 | 100.0 |
| 5-MSC | 178249 | 0.7 | 1 | 3.0 | n.d. | 3.0 |
| Nitidine chloride | 146397 | 0.001 | 1 | 12.0 | n.d. | 64.0 |
| Puromycin | – | 0.1 | 1 | 12.0 | 120.0 | 50.0 |
| Rhodamine 123 | – | 1.2 | 1 | 65.0 | n.d. | 75.0 |
| Rubidazone | 164011 | 0.005 | 1 | 8.5 | n.d. | 13.0 |
| Vinblastine | 49482 | 0.0007 | 1 | 11.5 | 100.0 | >20.0 |
| Vincristine | 67574 | 0.0015 | 1 | 10.0 | n.d. | 15.0 |
| Variamycin | 269146 | 1.2 | 1 | 12.0 | n.d. | 25.0 |
| VM26 | 122819 | 0.01 | 1 | 4.5 | 50.0 | 10.0 |
| VP16-213 | 141540 | 0.1 | 1 | 8.0 | 45.0 | 16.5 |
| Taxol | 125973 | 0.0015 | 1 | 25.0 | 200.0 | 35.0 |

[a]The $D_{10}$ value represents the concentration of the drug which reduces plating efficiency of a cell line to 10% of that observed in the absence of any drug. The $D_{10}$ values of various drugs for the HeLa and the mutant cell lines were obtained from experiments similar to those described in **Figure 1**. Similar results ($\pm 10\%$) with these cell lines have been obtained in at least 2 independent experiments. [b]Assuming the $D_{10}$ values of various drugs toward HeLa cells as 1, the relative degrees of resistance of the mutant cell lines were obtained from the ratios of the $D_{10}$ values of the mutant cell lines and the HeLa cells. [c]The $Pur^{RII}7$ cells which are maintained in medium containing $10\,\mu g\,ml^{-1}$ puromycin were grown for 3 weeks in the absence of puromycin before these experiments were carried out. The resistance pattern of $Pur^{RI}27$ is completely stable and is unaffected under these conditions.

employing a monoclonal antibody for the P-glycoprotein (kindly provided by Dr V. Ling, Ontario Cancer Institute) have failed to detect increased expression of this protein in these mutants (unpublished results) this may be due to the relatively low level of resistance of these mutant cells. To clarify this aspect, further studies using cloned cDNA probes for the MDR gene are being carried out.

The main emphasis of the current work, however, was on examining in detail the cross-resistance pattern of a human MDR cell line towards various anticancer drugs. In the past, only limited studies in this regard with the human MDR cell lines have been reported (Beck, 1983; Akiyama *et al.*, 1985; Twentyman *et al.*, 1986*a*, b). For many of the drugs employed in the present investigation (*viz.* Baker's antifol, chalcomycin, coralyne sulphoacetate, harringtonine, homoharringtonine, tamoxifen, reumycin and thalicarpine, etc.), the cross-resistance of the MDR cell line has been examined for the first time. Based on the cross-resistance pattern of the $Pur^R$ mutants, the drugs which have been examined could be divided into two groups: those to which the MDR mutants exhibit increased resistance (Table II) and the others for which no significant change in resistance is observed (Table III). The former group includes a number of antimitotic drugs (*viz.* colchicine, taxol, maytansine, vinblastine, vincristine), a few protein synthesis inhibitors (*viz.* puromycin, chalcomycin, harringtonine, homoharringtonine, bruceantin), several compounds which either directly bind or intercalate into DNA (*viz.* aclacinomycin A, actinomycin D, adriamycin, m-AMSA, chromomycin $A_3$, coralyne sulphoacetate, daunomycin, ellipticine, mithramycin, mitoxantrone, 5-MSC, nitidine chloride, rubidazone, variamycin, VM26 and VP16-213). A few other drugs to which the $Pur^R$ mutants exhibit cross-resistance (*viz.* rhodamine 123, Baker's antifol) may act via other mechanisms.

In an earlier study the cross resistance pattern of single-step mutants of Chinese hamster ovary (CHO) cells selected for resistance to either vinblastine or taxol to a large number of these drugs was examined (Gupta, 1985). A comparison of the results of these two studies indicates that the cross resistance patterns of these independently selected mutants resistant to various drugs (*viz.* taxol, vinblastine and puromycin) in cells of two different species, are virtually identical. The pattern observed here is also in accordance with the results of limited cross resistance studies carried out with various other MDR cell lines of different species reported in the literature (Dano, 1972; Skovsgaard, 1981; Beck, 1983; Biedler *et al.*, 1983; Ling *et al.*, 1983; Akiyama *et al.*, 1985; Twentyman *et al.*, 1986). These results provide strong evidence that the MDR mutants of different species exhibit increased resistance to the same specific group of drugs, listed in Table I.

The results presented in this paper have a number of important implications regarding clinical applications of antineoplastic drugs. Our studies provide evidence that mutants exhibiting MDR phenotype could be obtained in human cells after a single-step selection and that multiple selection or prolonged growth in selective medium is not necessary for generation of this phenotype. Although the frequency of resistant mutants in control HeLa cell population is low ($<1\times10^{-7}$) in a tumour that contains a large number of target cells ($\simeq 10^8$ or more), the resistant cells may pre-exist and their growth will be selectively enhanced in the presence

**Table III** Anticancer drugs to which $Pur^R$ mutants do not show cross resistance

| Drug | NSC no. | $D_{10}$ value for HeLa cells ($\mu g\, ml^{-1}$) | Relative degree of resistance[a] HeLa | $Pur^{RI}27$ | $Pur^{RII}7$ |
|---|---|---|---|---|---|
| Anguidine | 141537 | 0.006 | 1 | 1.0 | 0.8 |
| Asaley | 167780 | 0.004 | 1 | 1.0 | 1.0 |
| AT-125 | 163501 | 0.15 | 1 | 0.7 | 0.8 |
| 5-Azacytidine | 102816 | 0.12 | 1 | 0.6 | 0.6 |
| Azaserine | – | 0.05 | 1 | 1.0 | 1.0 |
| Bleomycin | 125066 | 5.0 | 1 | 1.0 | 1.0 |
| Chlorambucil | 3088 | 7.0 | 1 | 1.0 | 0.8 |
| Chlorpromazine | – | 3.5 | 1 | 1.0 | 1.0 |
| *Cis*-platin | 119875 | 0.3 | 1 | 1.0 | 0.8 |
| Cyclocytidine | 145668 | 0.01 | 1 | 1.0 | 0.7 |
| Cytosine arabinoside | 63878 | 0.025 | 1 | 1.0 | 0.8 |
| α-DFMO | – | 5.0 | 1 | 0.8 | 0.8 |
| 5-Fluorouracil | 19893 | 0.2 | 1 | 1.0 | 1.0 |
| Ftorafur | 148958 | 4.0 | 1 | 1.0 | 1.0 |
| Gallium nitrate | 15200 | 7.5 | 1 | 1.0 | 1.0 |
| Hydroxyurea | 32065 | 12.5 | 1 | 1.0 | 1.0 |
| ICRF-159 | 129943 | 35.0 | 1 | 1.0 | 1.0 |
| ICRF-187 | 169780 | 30.0 | 1 | 1.0 | 1.0 |
| Impiramine | – | 15.0 | 1 | 1.0 | 1.0 |
| Melphalan | 8806 | 10.0 | 1 | 1.0 | 1.0 |
| 6-MeMPR | – | 0.008 | 1 | 1.0 | 1.0 |
| Methotrexate | 740 | 0.007 | 1 | 1.0 | 1.0 |
| Mitomycin C | 26980 | 0.015 | 1 | 1.6 | 1.5 |
| Methyl GAG | 32946 | 0.15 | 1 | 1.0 | 1.0 |
| Mycophenolic acid | – | 0.05 | 1 | 0.8 | 1.0 |
| Nafoxidine HCl | 70735 | 2.0 | 1 | 1.0 | 1.0 |
| Reumycin | 99733 | 50.0 | 1 | 1.0 | 1.0 |
| 6-Selenoguanosine | 137679 | 0.25 | 1 | 1.0 | 1.0 |
| Tamoxifen | 180973 | 4.0 | 1 | 1.0 | 1.0 |
| Thalicarpine | 68075 | 7.0 | 1 | 1.0 | 1.0 |
| 6-Thioguanine | 752 | 0.03 | 1 | 1.0 | 1.0 |
| Tiapamil | – | 50.0 | 1 | 1.0 | 1.0 |
| Tiazofurin | 286193 | 2.5 | 1 | 1.0 | 1.0 |
| Verapamil | – | 25.0 | 1 | 1.0 | 1.0 |

[a]The relative resistances of the mutant cell lines as compared to the parental HeLa cells were determined as described in **Table II**.

**Table IV** Effect of verapamil on the drug resistance of the mutant lines

| Anticancer drug | Verapamil $\mu g\, ml^{-1}$ | $D_{10}$ values for the cell lines ($ml^{-1}$) HeLa | $Pur^{RI}27$ | $Pur^{RII}7$ |
|---|---|---|---|---|
| Puromycin | 0 | 0.12 μg | 1.2 μg | 5.5 μg |
| | 1 | 0.11 μg | 0.45 μg | 2.5 μg |
| | 3 | 0.12 μg | 0.30 μg | 0.40 μg |
| | 10 | 0.11 μg | 0.12 μg | 0.15 μg |
| Taxol | 0 | 2.0 ng | 50.0 ng | 70.0 ng |
| | 1 | 2.0 ng | 15.0 ng | 20.0 ng |
| | 3 | 2.0 ng | 7.0 ng | 8.0 ng |
| | 10 | 1.8 ng | 3.0 ng | 3.5 ng |
| Actinomycin D | 0 | 0.08 ng | 1.2 ng | 2.6 ng |
| | 10 | 0.05 ng | 0.05 ng | 0.06 ng |
| Bisantrene | 0 | 1.5 ng | 10.0 ng | 14.0 ng |
| | 10 | 1.5 ng | 1.5 ng | 1.5 ng |
| Daunomycin | 0 | 4.0 ng | 40.0 ng | n.d. |
| | 10 | 3.5 ng | 4.0 ng | n.d. |
| Mitoxantrone | 0 | 15.0 ng | 1000 ng | 2000 ng |
| | 10 | 15.0 ng | 30.0 ng | 40.0 ng |
| Vinblastine | 0 | 1.0 ng | 10.0 ng | 20.0 ng |
| | 10 | 0.4 ng | 0.4 ng | 0.6 ng |
| VP16-213 | 0 | 0.10 μg | 1.1 μg | 1.8 μg |
| | 10 | 0.10 μg | 0.13 μg | 0.2 μg |

The $D_{10}$ values (given in either $\mu g\, ml^{-1}$ or $ng\, ml^{-1}$) of the cell lines for various drugs were determined in parallel in presence of either the solvent control (i.e., O verapamil) or the indicated concentrations of verapamil. n.d. = not determined.

of the selective drugs. Since many of the antineoplastic drugs are mutagenic (Singh & Gupta, 1983), treatment with such drugs may result in both induction as well as selective growth of the resistant cells. In accordance with this expectation, cells exhibiting cross resistance to a variety of drugs have been reported in human tumour cell populations (Bech Hansen *et al.*, 1977; Shoemaker *et al.*, 1983). Recently, increased expression of P-glycoprotein or of the corresponding gene, in a number of human tumours which are refractory to chemotherapy, has also been reported (Bell *et al.*, 1985; Gerlach *et al.*, 1986; Fojo *et al.*, 1987). The observation that all of the refractory tumours do not show increased expression of the P-glycoprotein may be related to the lower degree of resistance of such tumours, similar to that seen with the present mutants.

Asssuming that the cells exhibiting MDR phenotype may either pre-exist or are readily induced/selected in a tumour cell population, and are the major impediment in the success of chemotherapy, two possible strategies could be employed to overcome clinical resistance. First, based on the cross resistance information regarding such mutants, more effective drug combinations to which cellular resistance would not readily develop (i.e., in a single selection step) could be formulated. Such a combination should include no more than one drug from the first group (listed in Table II, to which resistance develops simultaneously) and one or more agents from the second group (Table III), to which the mutants exhibiting MDR phenotype do not show increased resistance. Interestingly, the MDR mutants exhibit marginally enhanced sensitivity to a number of agents in the latter group (*viz*. AT-125, 5-azacytidine, α-DFMO; Table III), and the use of these drugs in combination with the first group of drugs may prove particularly effective in preventing the emergence of MDR cells. The second strategy to overcome drug resistance should make use of the observation that the MDR phenotype of human cells is completely reversed in the presence of agents such as verapamil. Besides verapamil, a large number of other compounds (e.g., reserpine, quinidine, trifluoperazine, chlorpromazine, chloroquine, etc.) have been reported to cause reversal of the MDR phenotype (Inaba *et al*., 1981; Tsuruo *et al*., 1981, 1984; Ganpathi & Grabowski, 1983; Beck, 1984; Akiyama *et al*., 1988). Although the mechanism by which these compounds cause reversal of the MDR is unclear at present, the use of one of these agents in combination with the drugs listed in Table I should enhance the chemotherapeutic effectiveness of the latter drugs. Based on the above considerations, more effective chemotherapeutic drug combinations in principle could be obtained by combining the above two strategies, i.e., employing a drug from the first group (Table II) in conjunction with an agent which overcomes MDR and one or more drugs from the second group (Table III), to which the MDR mutants do not exhibit cross resistance.

### References

AKIYAMA, S.-I., FOJO, A., HANOVER, J. & 2 others (1985). Isolation and genetic characterization of human KB cell lines resistant to multiple drugs. *Som. Cell Genet.*, **11**, 117.

AKIYAMA, S.-I., CORNWELL, M.N., KUWANO, M.M. & 2 others. (1988). Most drugs that reverse multidrug resistance also inhibit photoaffinity labeling of P-glycoprotein by a vinblastine analog. *Molecular Pharmacol.*, **33**, 143.

AMES, G.F (1986). The basis of multidrug-resistance in mammalian cells. Homology with bacterial transport. *Cell*, **47**, 323.

BECH-HANSEN, W.T., SARANGI, M.F., SUTHERLAND, D.J.A. & 1 other (1977) Rapid assay for evaluating the drug sensitivity of tumor cells. *J. Natl Cancer Inst.*, **59**, 21.

BECK, W.T. (1983), Vinca alkaloid-resistant phenotype in cultured human leukemic lymphoblasts. *Cancer Treat. Rep.*, **67**, 875.

BECK, W.T. (1984). Cellular pharmacology of vinca alkaloid resistance and its circumvention. *Adv. Enzyme Regulation*, **22**, 207.

BECK, W.T. (1987). The cell biology of multiple drug resistance. *Biochem. Pharmacol.*, **36**, 2879.

BELL, D.R., GERLACH, J.H., KARTNER, N. & 2 others (1985). Detection of p-glycoprotein in ovarian cancer: A molecular marker associated with multidrug resistance. *J. Clin. Oncology*, **3**, 311.

BIEDLER, J.L., CHANG, T., MEYERS, M.B. & 0 others (1983). Drug resistance in Chinese hamster lung and mouse tumor cells. *Cancer Treat. Rep.*, **67**, 859.

CASS, C.E. (1972). Density-dependent resistance to puromycin in cell cultures. *J. Cell Physiol.*, **79**, 139.

CREMISI, C., SONENSHEIN, G.E. & TOURNIER, P. (1974). Studies on the mechanism of actinomycin D resistance of an SV-40 transformed hamster cell line. *Exp. Cell. Res.*, **89**, 89.

DANO, K. (1972). Cross resistance between vinca alkaloids and anthracyclines in Ehrlich ascites tumor *in vivo*. *Cancer Chemother. Rep.*, **56**, 701.

DANO, K. (1973). Active outward transport of daunomycin in resistant Ehrlich ascites tumor cells. *Biochim. Biophys. Acta*, **323**, 466.

DEVITA, V.T. & SCHEIN, P.S. (1973). The use of drugs in combination for the treatment of cancer. *N. Engl. J. Med.*, **288**, 998.

FOJO, A.S., UEDAS, K., SLAMON, D.J. & 2 others (1987). Expression of a multidrug resistant gene in human tumors and tissues. *Proc. Natl. Acad. Sci. USA*, **84**, 265.

GANPATHI, R. & GRABOWSKI, D. (1983). Enhancement of sensitivity to adriamycin in resistant P388 leukemia by calmodulin inhibitor trifluoperazine. *Cancer Res.*, **43**, 3696.

GERLACH, J.H., KARTNER, N., BELL, D.R. & 1 other (1986). Multidrug resistance. *Cancer Surveys*, **5**, 25.

GOLDIE. J.H., COLDMAN, A.J. & GUDAUSKAS, G.A. (1982). Rationale for the use of alternating non-cross resistant chemotherapy. *Cancer Treat. Rep.*, **66**, 439.

GOLDIE, J.H. & COLDMAN, A.J. (1984). The genetic origin of drug resistance in neoplasm: Implications for systemic therapy. *Cancer Res.*, **44**, 3643.

GROS, P., BEN NERIAH, Y., CROOP, J.M. & HOUSMAN, D.E. (1986). Isolation and expression of a complementary DNA that confers multidrug resistance. *Nature*, **323**, 728.

GUPTA, R.S. (1983*a*). Podophyllotoxin resistant mutants of Chinese hamster ovary cells: Cross resistance studies with various microtubule inhibitors and podophyllotoxin analogs. *Cancer Res.*, **43**, 505.

GUPTA, R.S. 1983*b*). Genetic, biochemical and cross-resistance studies with mutants of Chinese hamster ovary cells resistant to anticanccer drugs VM-26 and VP16-213. *Cancer Res.*, **43**, 1568.

GUPTA, R.S. (1985). Cross-resistance of vinblastine and taxol-resistant mutants of Chinese hamster ovary cells to other anticancer drugs. *Cancer Treat. Rep.*, **69**, 515.

GUPTA, R.S., CHENCHAIAH, P.C. & GUPTA, R. (1987). Synthesis and structure activity relationships among glycosidic derivatives of 4-demethylepipodophyllotoxin and epipodophyllotoxin, showing VM26- and VP16-213-like activities. *Anticancer Drug Design*, **2**, 1.

HILL, B.T. (1984). *Collateral Sensitivity and Cross Resistance in Antitumour Drug Resistance*. Fox, B.W. and M. Fox (eds) Ch. 24, 673. Springer-Verlag, Berlin.

INABA, M. & JOHNSON, R.K. (1977). Decreased retention of actinomycin D as the basis for cross-resistance in anthracycline-resistant sublines of P388 leukemia. *Cancer Res.*, **37**, 4629.

INABA, M., FUJIKURA, S., TSUKAGOSHI, S. & 1 other (1981). Sensitivity of adriamycin and vincristine resistant P388 leukemia restored *in vitro* with reserpine. *Biochem. Pharmacol.*, **90**, 2191.

KESSEL, D. & WILBERDING, C. (1985). Anthracycline resistance in P388 murine leukemia and its circumvention by calcium antagonists. *Cancer Res.*, **45**, 1687.

LIEBERMAN, I. & OVE, P. (1959). Isolation and study of mutants from mammalian cells in culture. *Proc. Natl Acad. Sci. USA*, **45**, 867.

LING, V., KARTNER, N., SUDO, T. & 2 others (1983). Multidrug-resistant phenotype in Chinese hamster ovary cells. *Cancer Treat. Rep.*, **67**, 869.

LOUIE, K.G., HAMILTON, T.C., WINKER, M.A. & 8 others (1986). Adriamycin accumulation and metabolism in adriamycin-sensitive and resistant human ovarian cell lines. *Biochem. Pharmacol.*, **35**, 467.

MEYERS, M.B., SPENGLER, B.A., CHANG, T.D. & 2 others (1985). Gene amplification-associated cytogenetic observations and protein changes in vincristine resistant Chinese hamster, mouse and human cells. *J. Cell. Biol.*, **100**, 588.

MORROW, J., SAMMONS, D. & BARRON, E. (1980). Puromycin resistance in Chinese hamster cells: Genetic and biochemical studies of partially resistant, unstable clones. *Mutation. Res.*, **69**, 333.

RIORDAN, J.R., DEUCHARS, K., KARTNER, N. & 3 others (1985). Amplification of P-glycoprotein genes in multidrug-resistant mammalian cell lines. *Nature*, **316**, 817.

RIORDAN, J.R. & LING, V. (1985). Genetic and biochemical characterization of multidrug resistance. *Pharmacol. Ther.*, **28**, 51.

ROGAN, A.M., HAMILTON, T.C., YOUNG, R.C. & 2 others (1984). Reversal of adriamycin resistance by verapamil in human ovarian cancer. *Science*, **224**, 994.

RONINSON, I.B., ABELSON, H.T., HOUSMAN, D.E. & 2 others (1984). Amplification of specific DNA sequences correlates with multidrug resistance in Chinese hamster cells. *Nature*, **309**, 626.

SCHABEL, F.M., SKIPPER, H.E., TRADER, M.W. & 3 others (1983). Establishment of cross-resistance profiles for new agents. *Cancer Treat. Rep.*, **676**, 905.

SHOEMAKER, R.H., CURT, G.A. & CARNEY, D.N. (1983). Evidence for multidrug-resistant phenotype in human tumor cell populations. *Cancer Treat.*, **67**, 883.

SINGH, B. & GUPTA, R.S. (1983). Mutagenic responses of thirteen anticancer drugs on mutation induction at multiple genetic loci and on sister chromatid exchanges in Chinese hamster ovary cells. *Cancer Res.*, **43**, 577.

SINGH, B. & GUPTA, R.S. (1985). Species-specific differences in the toxicity and mutagenicity of the anticancer drugs mithramycin, chromomycin $A_3$ and olivomycin. *Cancer Res.*, **45**, 2813.

SKOVSGAARD, T. (1978). Mechanism of cross-resistance between vincristine and daunomycin in Ehrlich ascites tumor cells. *Cancer Res.*, **38**, 4722.

SLATER, L.M., MURRAY, S.L., WETZEL, M.W. & 2 others (1982). Verapamil restoration of daunorubicin responsiveness in daunorubicin-resistant Ehrlich ascites cells. *J. Clin. Invest.*, **70**, 1131.

TSURUO, T., ZIDA, H., TSUKAGOSHI, S. & SAKURAI, Y. (1981). Overcoming of vincristine resistance in P388 leukemia *in vivo* and *in vitro* through enhanced cytotoxicity of vincristine and vinblastine by verapamil. *Cancer Res.*, **41**, 1967.

TSURUO, T., IIDA, H., KITATANYI, Y. & 3 others (1984). Effects of quinidine and related compounds on cytotoxicity and cellular accumulation of vincristine and adriamycin in drug resistant tumor cells. *Cancer Res.*, **44**, 4303.

TWENTYMAN, P.R., FOX, N.E., WRIGHT, K.A. & 1 other (1986*a*). Derivation and preliminary characterization of adriamycin resistant lines of human lung cancer cells. *Br. J. Cancer*, **53**, 529.

TWENTYMAN, P.R., FOX, N.E. & BLEEHEN, N.M. (1986*b*). Drug resistance in human lung cancer cell lines: Cross-resistance studies and effects of the calcium transport blocker, verapamil. *Int. J. Radiat. Oncol. Biol. Phys.*, **12**, 1355.

WARR, J.R., BREWER, F., ANDERSON, M. & FERGUSSON, J. (1986). Verapamil hypersensitivity opf vincristine-resistance Chinese hamster ovary cell lines. *Cell. Biol. Int. Rep.*, **10**, 389.